# Conference Report: THIRD INTERNATIONAL CONFERENCE ON CHEMICAL KINETICS REACTIONS IN GAS AND CONDENSED MEDIA Gaithersburg, MD July 12–16, 1993

**DOI:** 10.6028/jres.098.048

**Published:** 1993

**Authors:** Robert E. Huie

**Affiliations:** Chemical Kinetics and Thermodynamics Division, National Institute of Standards and Technology, Gaithersburg, MD 20899-0001

## 1. Introduction

This quadrennial conference is organized to bring together leading scientists who generate and use chemical kinetic data in both the gas and condensed phases. Chemical kinetics is increasingly used as a tool for the understanding and control of complex chemical processes in a number of different areas. Many reaction types and intermediates are of key importance under widely different conditions. Nevertheless, investigators in one discipline are frequently unaware of work carried out in another area which may be related to their work, particularly if the other work is carried out in a different physical phase. This conference brings together scientists from these different disciplines to discover areas of common interest where knowledge from one area can illuminate problems of another.

The conference was supported by the National Institute of Standards and Technology, the Environmental Protection Agency, the National Aeronautics and Space Administration, the NIST Standard Reference Data Program, the Chemical Manufacturers Association, and the Exxon Research and Engineering Company. Support was also provided in the form of special travel grants to attend this conference given by the International Science Foundation to some participants from the former Soviet Union. During the conference, the NIST Standard Reference Data Program demonstrated some of their computer databases and provided access to electronic mail for the participants.

Over two hundred scientists registered to attend the conference. They included representatives from over 20 countries. The delegates heard 44 oral presentations, including 6 invited lectures, on a wide range of subjects. Also, there were about 140 poster presentations.

## 2. Proceedings

The conference opened with a broad session titled Decomposition Kinetics chaired by John T. Herron of NIST. Jürgen Troe of the University of Göttingen delivered an invited talk on theoretical methods for the treatment of thermal high pressure dissociation and recombination reactions. Subsequent talks in this session and in the afternoon poster session dealt with the experimental determisession on Heterogeneous Reactions, chaired by Pedatsur Neta of NIST, started with an invited talk by Janos H. Fendler of Syracuse University on microheterogeneous systems, a topic which was new to most of the participants. The other talks in that session were on reactions at gas-solid or gas-liquid interfaces, while various poster presentations also addressed micellar reactions.

On the second morning, the session on Theory, chaired by Wing Tsang of NIST, opened with an invited lecture by Carl F. Melius of Sandia National Laboratory in which the application of the BAC-MP4 method to the calculation of the thermochemical properties of reaction intermediates was outlined. A large number of heats of formation of reaction intermediates have been calculated in this manner. Because of a large number of requests, the Conference arranged to have a large table of these results duplicated and made available to the conferees. Subsequent talks in the morning session, and many of that day’s poster presentations, expanded considerably on this theme and on other theoretical approaches to chemical kinetics. These methods are proving to be very important as guidance to experimental work and in the analysis of experimental data.

The Tuesday afternoon session, chaired by Arthur Fontijn of Rensselaer Polytechnic Institute, started with an invited talk by James C. Weisshaar of the University of Wisconsin on the reactions of gas-phase metal atoms and ions with alkenes and alkanes. The reactivity of the metal atoms and ions correlate well with the known atomic energy levels and with simple models of metal-hydrocarbon chemical bonding. Few of the participants were aware of the extent to which these studies have been carried out. The subsequent talks in the afternoon session focussed on reactions of silicone-containing radicals, as did many of the posters. A number of other poster presentations were on other aspects of the kinetics of chemical vapor deposition, which is becoming one of the important applications of chemical kinetics.

The Wednesday morning session opened with an invited presentation by Sidney W. Benson of the University of Southern California on the reaction of ethyl radicals with molecular oxygen. This session was chaired by Jai P. Mittal of the Bhabha Atomic Research Centre. Subsequent talks dealt further with the reactions of alkyl and other organic radicals with oxygen, particularly at high temperatures, as did several of the poster presentations that afternoon. In one of the papers, Dennis Bogan of Catholic University, presented results on the oscillating chemiluminescence observed from the reaction of methyl radicals with oxygen. Other papers were on the reactions of the peroxyl radicals formed from the alkyl radical-oxygen reaction.

The afternoon session was the first of two, the other being Thursday afternoon, to focus on the reactions of inorganic radicals. The Wednesday afternoon session was chaired by Jeffrey W. Hudgens and the Thursday afternoon session was chaired by Stephen E. Stein, both of NIST. Many of the poster presentations also involved reactions of inorganic radicals and much of the theoretical work discussed earlier in the meeting had to do with reaction pathways in these systems.

Complex organic reactions were the theme of the Thursday morning session, chaired by Michael J. Kurylo of NIST. The session opened with an invited talk on the kinetics and mechanisms of the reactions of organic peroxyl radicals by Timothy J. Wallington of Ford Motor Company. The session had a particularly strong atmospheric bent, with, for example, presentations on the reactions of the hydroxyl radical-aromatic adduct with nitrogen oxides and on the effect of water vapor on ozonolysis reactions. The atmospheric emphasis continued on Friday morning with a session on abstraction reactions of the hydroxyl radical, chaired by Robert F. Hampson of NIST. Several of these papers, and a number of the poster presentations Thursday afternoon, were on reactions of hydroxyl radicals with partially halogenated compounds being considered as replacements for the fully halogenated compounds, which are currently used as refrigerants, foam-blowing agents, solvents, and in other applications. This reaction is the major tropospheric loss mechanism for these species.

## 3. Conclusion

The emphasis on application in the field of chemical kinetics in general and in this conference in specific was reflected in the papers presented. The theoretical calculation of the properties of reaction intermediates are carried out because of the importance of these intermediates in practical problems; estimation techniques are developed to allow preliminary decisions to be made about industrially important chemicals; rate constant evaluations are driven by the needs of modelers; finally, the choice of reactions to be studied is frequently dictated by the immediate practical needs. Because of this, the chemical kinetics community is fragmented into small groups which interact more with their specific user community: in combustion, stratospheric chemistry, tropospheric chemistry, chemical vapor deposition, etc. This meeting provided the opportunity for kineticists to interact with kineticists in many different areas of research and to learn the progress that has been made in measurements and techniques.

A limited number of copies of the meeting abstract book are available from the conference chairman, Robert E. Huie.

